# Interaction between purinergic signalling and purinergic modulators with skin wound repair effectors: a systematic review of preclinical in vivo models

**DOI:** 10.1007/s11302-026-10178-y

**Published:** 2026-08-31

**Authors:** Silvânia Mól Pelinsari, Reggiani Vilela Goncalves, Patricia da Silva Mattosinhos, Mariáurea Matias Sarandy, Emerson Ferreira Vilela, Rômulo Dias Novaes

**Affiliations:** 1 Department of General Biology, Federal University of Vicosa, Vicosa, MG 36570-900 Brazil; 2 Plants for Human Health Institute, North Carolina Research Campus, NC State University, 600 Laureate Way, Kannapolis, NC 28081 USA; 3 Fundação Percival Farquhar - UNIVALE, Núcleo da Saúde, Curso de Biomedicina, Governador Valadares, MG Brazil; 4 Minas Gerais Agricultural Research Agency (Epamig Sul), Experimental Field of São Sebastião Do Paraíso, São Sebastião Do Paraíso, MG 37959-899 Brazil; 5 Institute of Biomedical Sciences, Department of Structural Biology, Federal University of Alfenas, Alfenas, MG 37130-001 Brazil; 6 Departament of Animal Biology, Federal University of Viçosa, Viçosa, 36570-900 Brazil

**Keywords:** Experimental pathology, Purinergic signalling, Tissue repair, Wound healing

## Abstract

Purinergic signalling plays a central regulatory role on cell proliferation, migration, differentiation, survival, and inflammation. However, the impact of purinergic modulators-based therapies on tissue repair is still overlooked. By using a systematic review framework, we investigated the therapeutic relevance of purinergic agonists and antagonists in animal models of skin wound healing. Following PRISMA guidelines, PubMed/Medline, Scopus, Web of Science, Embase, and CINAHL databases were searched, identifying 12 in vivo preclinical studies. Risk of bias was assessed using the SYRCLE tool. Topical or oral administration of CGS-21680 (4-[{*N*-ethyl-5*’*-carbamoyladenos-2-yl}aminoethyl] phenylpropionic acid), UTP (Uridine-5'-triphosphate), NECA (5'-N-ethylcarboxamidoadenosine), ATP (adenosine triphosphate), PDRN (polydeoxyribonucleotide), ADP (Adenosine-5'-diphosphate) and TMPS (Thymidine 5'-O-monophosphorothioate) agonists stimulated wound contraction, fibroblast and keratinocyte proliferation, angiogenesis, and collagen biosynthesis. These effects were associated with activation of Nrf2 (Nuclear factor erythroid 2–related factor 2), MAPK (Mitogen-Activated Protein Kinase), and ERK/CREB pathways, inflammation and oxidative/nitrosative stress down-regulation, and increased anti-inflammatory/regulatory cytokines (IL-10, IL-13, TGF-β) and growth factors (FGF, EGF). Conversely, the purinergic antagonists DMPX (3,7-dimethyl-1-propargylxanthine), CSC (8-(3-chlorostyryl) caffeine), SUR (Suramin), ENP (enprofylline), caffeine, CLOP (clopidogrel), MRS 2179 (2′-deoxy-N6-methyladenosine-3′,5′-bisphosphate), and MRS 2395 (2-Chloro-3′,5′-O-(benzoyl-β,γ-methylene) adenosine 5′-triphosphate), as well as genetic knockout for purinergic receptors delayed wound closure by inhibiting these pathways, impairing angiogenesis, collagenogenesis, and aggravating inflammation and oxidative damage. Purinergic agonists were associated with improved wound healing through inflammatory and oxidative modulation, enhanced angiogenesis, increased cell proliferation, and accelerated wound closure, whereas antagonists or genetic deficiency of purinergic receptors impaired tissue repair. This review was registered in the PROSPERO database (CRD420251010254).

**Graphical abstract:**

Overview of the effects of purinergic signalling modulation on cutaneous wound healing in murine models. Purinergic receptor activation by agonists accelerates wound closure, enhances cell proliferation and angiogenesis, and attenuates oxidative stress and pro-inflammatory cytokine production through activation of the PKA/ERK/CREB pathway. Conversely, receptor inhibition by antagonists or genetic deletion (KO) disrupts downstream signalling, resulting in delayed wound healing and increased inflammatory and oxidative damage.

**Figure:**
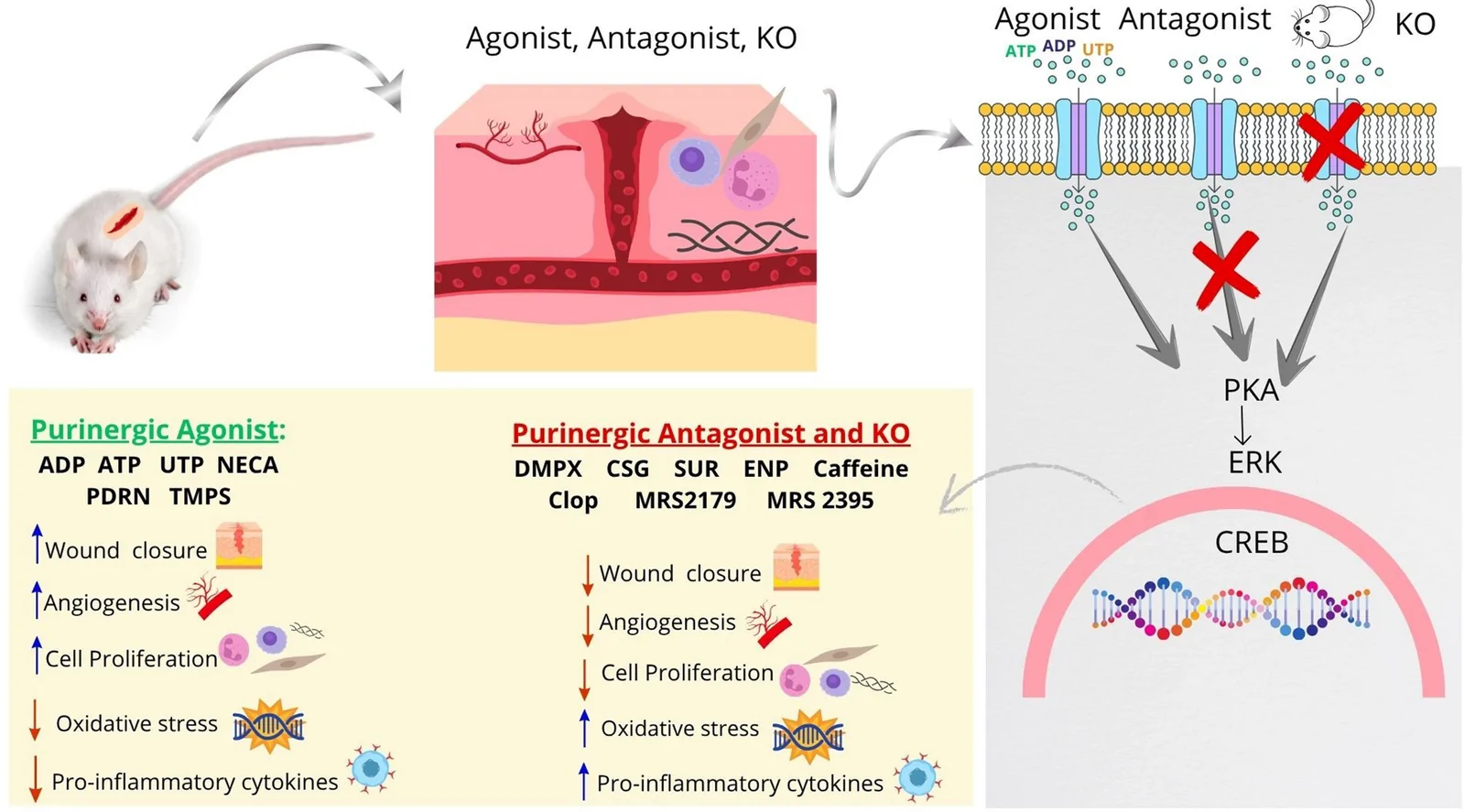

**Supplementary Information:**

The online version contains supplementary material available at https://doi.org/10.1007/s11302-026-10178-y.

## Introduction

Skin wounds affect a significant portion of the world’s population, representing a public health problem and substantial costs to the health system [1–3]. The global wound care market was valued at approximately USD 22.3 billion in 2023 and is projected to reach USD 31.7 billion by 2032 [4]. Wound repair is complex and involves several interdependent processes, whose failure in one or more of them can favor wounds chronicity and infections [5]. After injury, coagulation and hemostasis occurs and inflammatory cells recruitment is activated by pathogen-(PAMPs) and damage-(DAMPs) associated molecular patterns [5]. Then, fibroblasts, keratinocytes, and endothelial cells proliferate and synthesize extracellular matrix components such as fibronectin, collagen and elastic fibers [6, 7]. Simultaneously, angiogenesis is activated, supporting granulation tissue formation, which is gradually replaced by a thicker and more resistant tissue as it remodels [6, 7].

Currently, therapeutic strategies that accelerate wound healing are becoming increasingly relevant, and purinergic signalling pathways have emerged as promising targets in regenerative medicine [8]. Accordingly, there is evidence that adenosine nucleotides (AMP, ADP and ATP) are released in the extracellular compartment in response to injury, hypoxia and cell death, and their rapid conversion into adenosine activates different purinergic receptors, modulating events centrally involved in tissue repair such as inflammation, cell proliferation, migration, differentiation, and angiogenesis [8–11].

Purinergic signalling involves P1 (selective for adenosine) and P2 (selective for adenosine 5'-triphosphate [ATP], adenosine 5'-diphosphate [ADP], and uridine 5'-triphosphate [UTP] receptors [8]. P1 receptors are subdivided into 4 classes (A_1_, A_2_A, A_2_B, and A_3_) and modulate the activity of adenylate cyclase in an inhibitory (A_1_, A_3_) or stimulatory (A_2_A, A_2_B) manner, modulating cyclic adenosine monophosphate (cAMP) levels [8, 12, 13]. In addition, P2 receptors are subdivided in P2X and P2Y. P2X receptors are ligand-activated ion channels and are activated by extracellular ATP to induce Na^+^, K^+^, and Ca^2+^ flow across the plasma membrane. P2Y receptors belong to the G-protein-coupled superfamily [8, 12, 13].

Studies indicate that therapies based on purinergic agonists can accelerate skin repair with a low incidence of side effects [14, 15]. Accordingly, there is evidence that P2Y and P2X activation regulates ATP and adenosine dynamics, triggering intracellular cascades crucial for wound healing [16–18]. In turn, these mediators regulate MAPK/ERK1/2, p38, cAMP, and Nrf2 pathways, promoting antioxidant and anti-inflammatory effects, cell proliferation, angiogenesis, fibroblast migration, and collagen biosynthesis [19–21].

Despite these promising effects, there is still no clear understanding of which molecules, doses, duration, and routes of administration may be associated with the positive and negative therapeutic effects of purinergic modulators on skin wound repair [17, 22]. Therefore, we used a systematic review framework to analyze the preclinical in vivo evidence on the impact of purinergic agonists and antagonists, as well as deletion of genes encoding purinergic receptors on the healing of non-infected skin wounds. A mechanistic focus on specific purinergic pathways that regulate scar tissue microstructure, inflammation, and oxidative stress was adopted. In addition, we critically assessed the quality of the current evidence, highlighting the main limitations that must be overcome in further investigations.

## Methods and materials

### Focus question and protocol registration

The PICOS (population, intervention, comparison, outcomes) strategy was used to define the guiding question and the search strategy used in the systematic review. Accordingly, this systematic review sought to answer the following question: Do animals treated with modulators of the purinergic signalling pathway exhibit differences in skin wound healing indicators compared to animals not treated with these modulators?

Additionally, we investigated the impact of purinergic agonists and antagonists on mechanisms that control inflammation, redox metabolism, cell proliferation, neoangiogenesis, and collagenogenesis, which are centrally associated with the scarring process [22–26]. The protocol for this systematic review has been registered in PROSPERO (CRD420251010254) and is available at: https://www.crd.york.ac.uk/PROSPERO/view/CRD420251010254.

### Literature search strategy

**Fig. 1:**
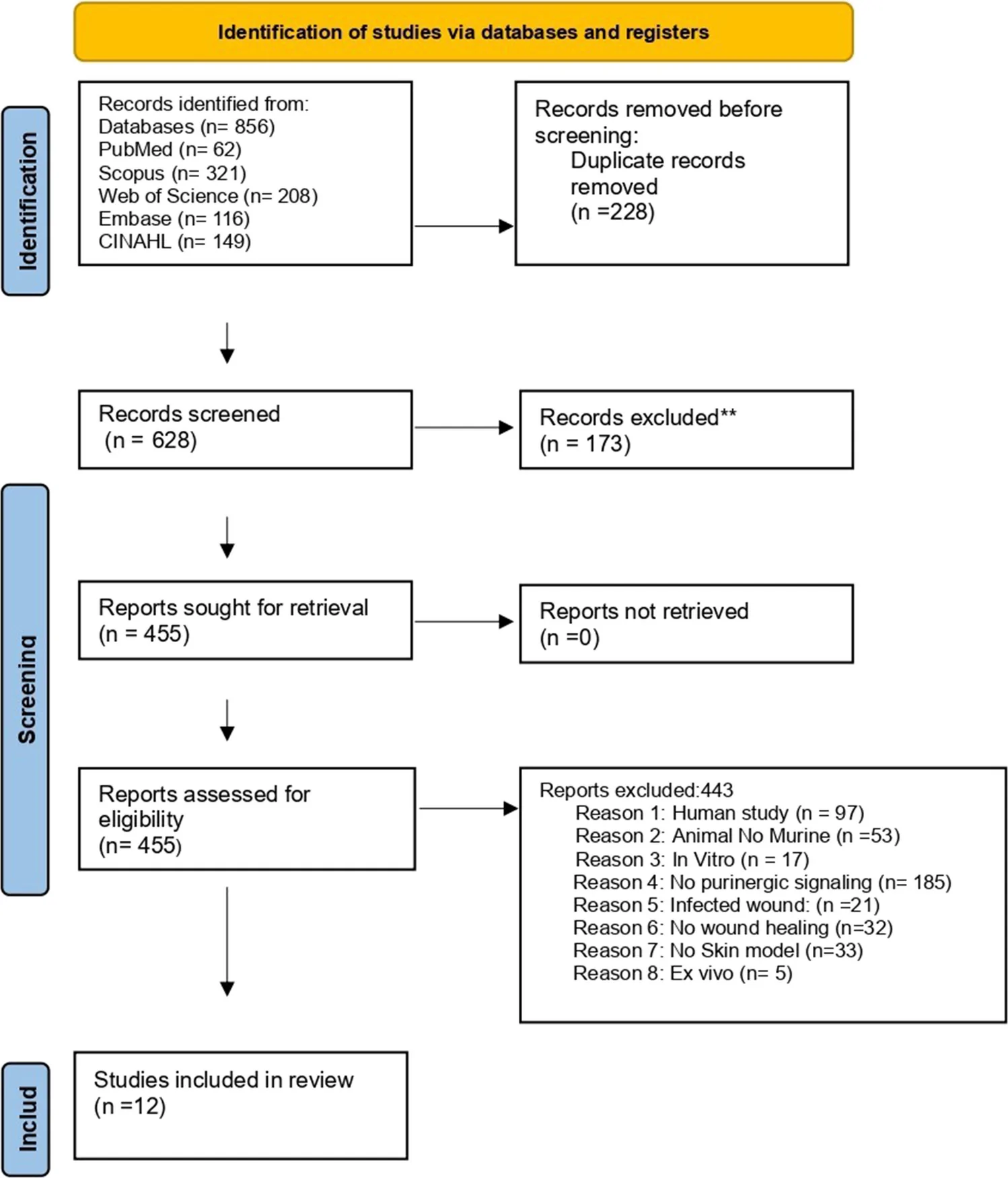
The Preferred Reporting Items for Systematic Reviews and Meta-Analyses (PRISMA) flow diagram. The flowchart indicates the research records obtained at all stages of the search process required to develop systematic reviews and meta-analyses. Based on the PRISMA Statement (http://www.prisma-statement.org)

This systematic review was conducted following the Preferred Reporting Items for Systematic Reviews and Meta-Analyses (PRISMA) guidelines [27], which guided studies  selection, screening, and eligibility (Fig. 1). An extensive bibliographic search was conducted using the electronic databases Medline/PubMed (https://www.ncbi.nlm.nih.gov/pubmed), Scopus (https://www.scopus.com/home.uri), Web of Science (https://www-webofscience-com.ez35.periodicos.capes.gov.br), Embase (https://www-embase-com.ez35.periodicos.capes.gov.br) on December 9, 2024 at 5:02 PM, and CINAHL (EBSCO) (https://research-ebsco-com.ez35.periodicos.capes.gov.br/c/iypcif/search). The search was completed on April 3, 2025 at 10:30 PM.

For all databases, the search filters were based on three levels: (i) Purinergic signalling, (ii) Wound Healing, and (iii) Skin, combined using Boolean connectors (OR/AND). In PubMed/Medline, standardized descriptors from the Medical Subject Headings (MeSH) database were used in combination with Title and Abstract (TIAB) algorithm to enhance retrieval efficiency. The search matrix was adapted for Scopus using the TITLE-ABS-KEY algorithm, for Web of Science using the TS- command, for Embase using exp algorithm and for CINAHL (EBSCO) using the AB command.

The following search terms and their combinations were used in all databases: “Purinergic signalling”, “Purinergic receptor*”, “Adenosine receptor*”, “Adenosine diphosphate”, “Adenosine triphosphate”, “Purinergic Agonist*”, “Purinergic Antagonists*”, “Purine*”, “Adenine”, “Guanine”, “Hypoxanthine”, “Xanthine”, “Theobromine”, “Caffeine”, “Uric acid”, “Isoguanine”, “Wound Healing*”, “Skin”, “Dermis”, “Granulation tissue”, “Epidermis”, “Keratinocytes”, “Integumentary system”, “Dermatology”, “Dermoscopy”, “Wounds and Injuries”, “Fibrosis”, “Scar*”, “Cicatrix”. A detailed description of the search strategy is available in the supplementary file (Table S1). No chronological limit was applied. Research records published in English, Portuguese, and Spanish were admitted.

Two reviewers (SMP and MMS) conducted the bibliographic search, removed duplicate articles, and read titles and abstracts to identify potentially relevant research records. After this initial selection, full-text articles were independently assessed for eligibility. The selections were then compared, and inconsistencies were resolved by arbitration by two expert researchers (RVG and RDN).

### Eligibility criteria

In the eligibility phase of the PRISMA guideline, only studies that met the following eligibility criteria were selected: (a) In vivo studies investigating the impact of purinergic modulation (chemical [purinergic agonists/antagonists] and/or genetic [gene knockout]) on the healing of non-infected cutaneous wounds; (b) studies including control wild-type animals with non-infected cutaneous wounds untreated. Studies were ineligible when exhibiting the following characteristics: (a) secondary studies (literature reviews, letters to the editor, commentaries, and editorials), (b) qualitative research, (c) in vitro or ex vivo studies, (d) research investigating infected wounds, (e) studies without an untreated control group, (f) unrelated to skin wound healing, (g) gray literature. The inter-rater agreement obtained through our search strategy was evaluated using the kappa coefficient [28].

### Data extraction and synthesis

Research data were extracted from all eligible studies by three independent reviewers (SMP, MMS, and RDN) in a systematic way, using standardized extraction masks delimiting the following information: (a) Publication characteristics (author, country, and year). (b) Animal model characteristics (species, sex, weight, age, number of animals per group, gene knockout model, control group, and ethics committee approval). (c) Treatment information (drug used [purinergic agonist or antagonist], dose, frequency, period, and administration route). (d) Primary outcomes (wound size and closure time/rate, cell proliferation indexes, angiogenesis, and collagen content. (e) Secondary outcomes: inflammatory and oxidative stress markers. Missing data were requested from the authors via e-mail. Discrepancies in data extraction by at least two of the three reviewers were verified and corrected by a fourth expert reviewer (RVG).

### Bias analysis

The methodological quality of the included studies was assessed using the risk of bias (RoB) statement, a tool from the Systematic Review Centre for Laboratory Animal Experimentation (SYRCLE), specifically designed for animal studies [29]. This instrument is based on the Cochrane Collaboration tool for assessing the risk of bias in randomized trials and is adjusted for bias aspects that play a specific role in animal intervention studies. To increase transparency and applicability, standardized signalling questions were used to guide researchers’ judgment, based on the following domains**:** selection bias assessed random sequence generation baseline characteristics, and allocation concealment to ensure equivalent groups at baseline; performance bias focused on random assignment and blinding of caregivers and researchers to minimize conscious or subconscious bias; detection bias analyzed random outcome assessment and blinding to ensure objective and consistent outcome measurement. Attrition bias involves the assessment of incomplete outcome data, as high dropout rates can lead to biased results. Other bias components were related with limited information on treatment protocols, ethical approval, validated tools, and statistical methods. We used the Cochrane Review Manager 5.3 (RoB 2.0) program to construct a figure visually presenting the risk of bias across all studies reviewed. The items in the RoB tool were categorized as “yes” (low risk of bias), “no” (high risk of bias), or “unclear” (not reported, making the risk uncertain). This comprehensive approach ensures that conclusions are based on solid and reliable data, contributing to more effective and ethical scientific research.

## Results

### Research records and studies reviewed

Our search strategy retrieved a total of 856 studies (PubMed: 62, Scopus: 321, Web of Science: 208, Embase: 116, CINAHL: 149). After removing 228 duplicates, 173 studies were excluded due to inappropriate topics based on title and abstract screening. Then, 455 studies were fully reviewed (full-text reading), and 443 were excluded based on eligibility criteria, as they involved in vitro models, studies unrelated to purinergic signalling, infected wounds, studies unrelated to skin wound healing, ex vivo models, or human studies. This resulted in a final selection of 12 eligible studies (Fig. 1; Table S2), with a 0.908 kappa coefficient, indicating a substantial inter-rater agreement in the selection process.

### Characteristics of the animal models

The general characteristics of the selected studies and experimental models are shown in Tables S2 and S3. The 12 studies were published between 1997 and 2024 and were conducted in eight different countries: United States (42%, *n* = 5), China, Brazil, Germany, India, Japan, Poland, and South Korea (8%, *n* = 1 each) (Figure S1).

**Fig. 2:**
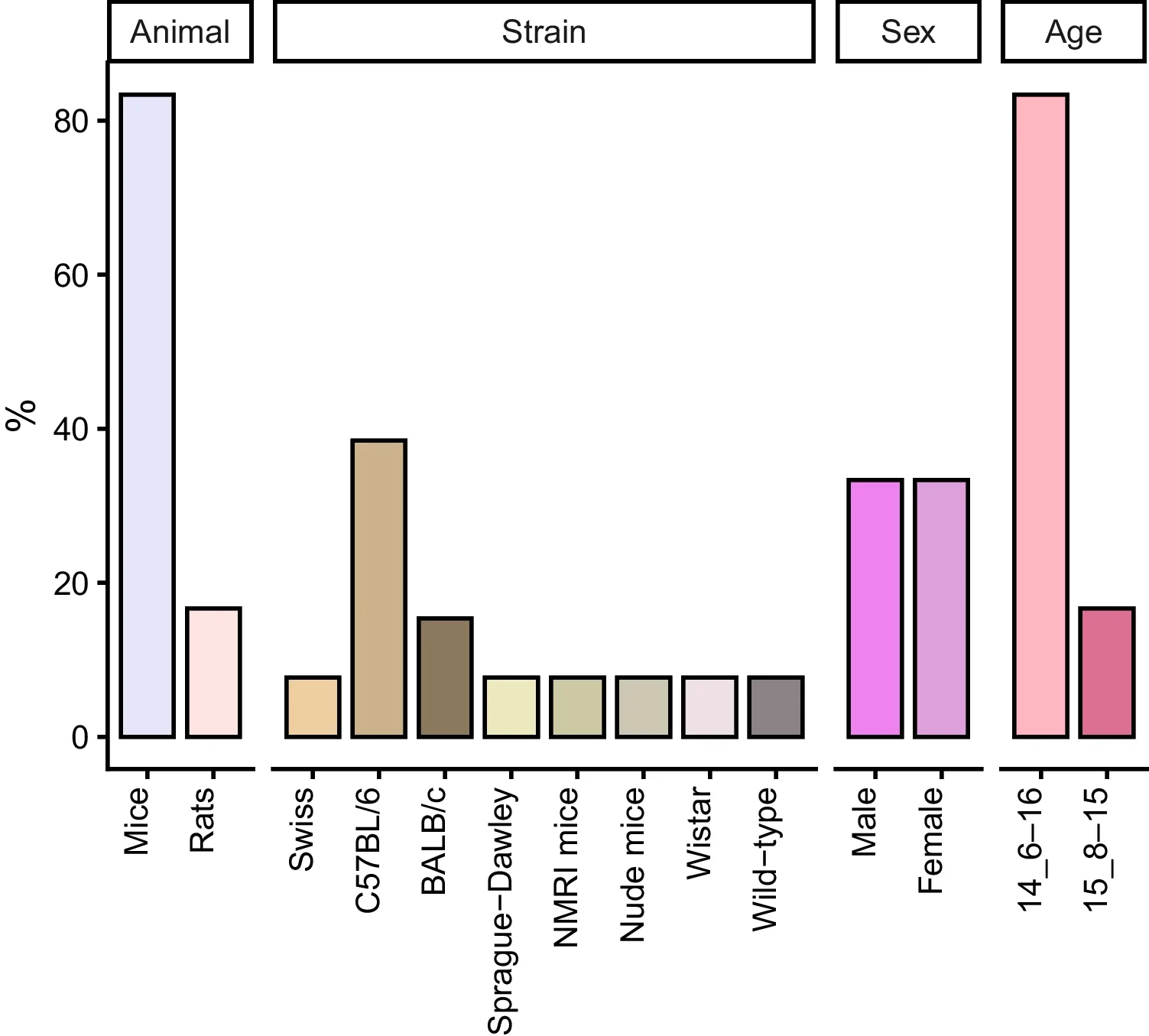
Animal model characteristics. Overview of species, lineages, sex distribution, and age ranges of animals used in the included in vivo studies, illustrating murine models and the diversity of mouse and rat lineages. Additional details are shown in Table S3. Refer to supplementary material

**Fig. 3:**
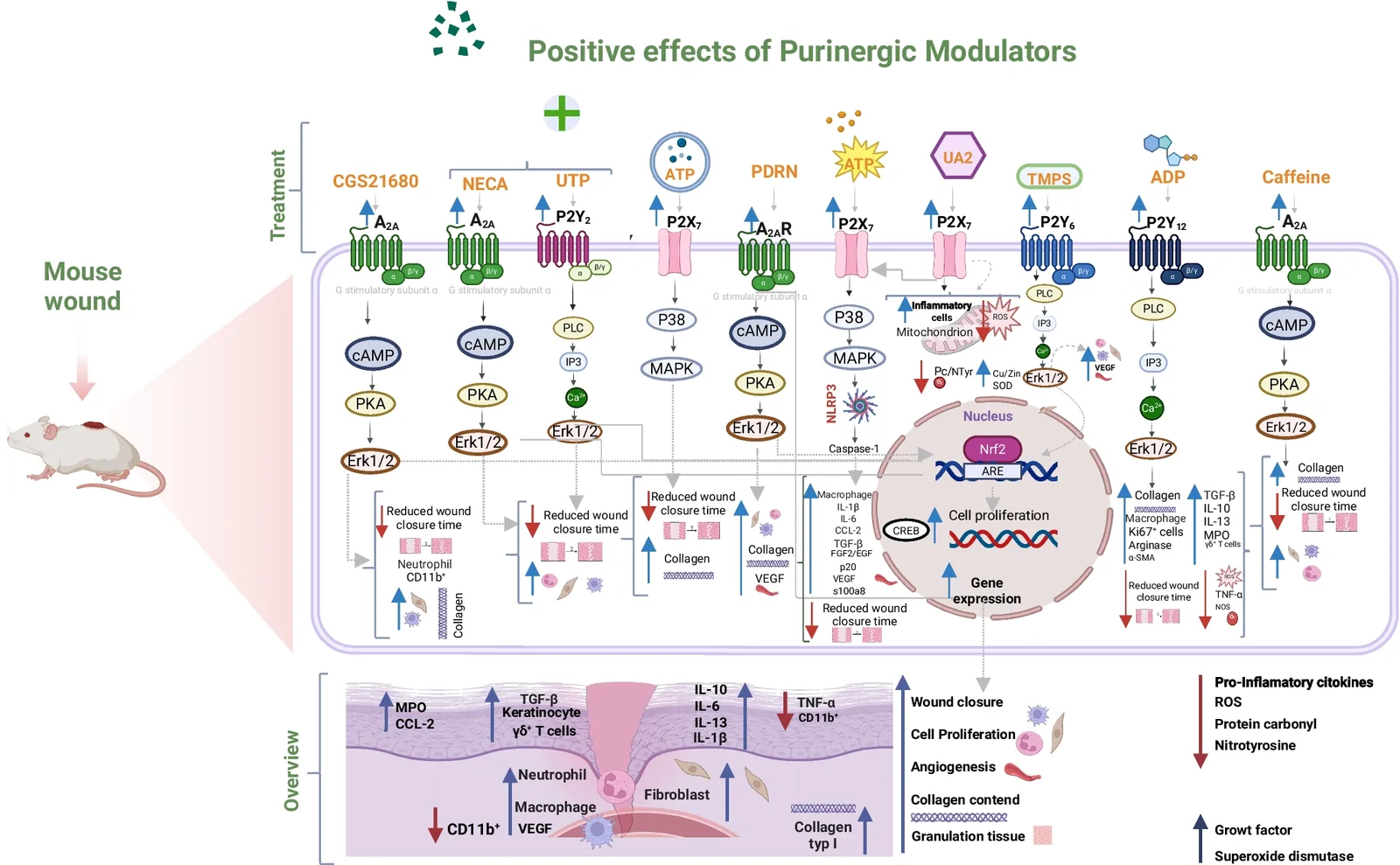
Positive effects of purinergic modulators on wound healing in a murine model. Treatment with adenosine A_2_A receptor agonists (CGS21680), ATP, adenosine, PDRN, NECA, UTP, TMPS, ADP, and uric acid activates P2X and P2Y receptors, triggering MAPK, cAMP, and ERK/CREB signalling. These pathways induce NRF2 activation, regulating genes involved in cell proliferation, tissue repair, inflammation control, and antioxidant defense. Purinergic modulation reduces pro-inflammatory cytokines (TNF-α) and increases anti-inflammatory (IL-10, IL-13) and regulatory (TGF-β) cytokines, promoting a healing microenvironment. Consequently, fibroblast migration, collagen deposition, extracellular matrix remodeling, angiogenesis via VEGF induction, and wound closure are enhanced, while inflammation and oxidative stress markers (MPO, nitrotyrosine, iNOS, protein carbonylation) are reduced and SOD activity is increased, collectively supporting efficient tissue regeneration

Mice were the main animal model used in the studies (*n* = 10; 83.3%), followed by rats (*n* = 2; 16.6%). C57BL/6 (*n* = 5; 41.6%) and Balb/c (*n* = 2; 16.6%) were the main mice lineage used (Fig. 3, Table S3), while Wistar 8% (*n* = 1) and Sprague–Dawley (*n* = 1; 8.3%) were the rats lineage used. NMRI, Nude, and Swiss mice were reported in 1 (8%) study each. Male (n = 5; 41.6%) and female (n = 4; 33.3%) animals were included in unequal proportions (Fig. 2, Table S3). Male (*n* = 5; 41.7%) and female (*n* = 4; 33.3%) animals were included, but sex was not reported in five studies (41.7%). Rats age ranged from 8–15 weeks-old.

### Skin wound characteristics

Excisional wounds were predominant, accounting for n = 11 studies (92%). Only one study involved incisional wounds (*n* = 1, 8%). Among the studies conducted in mice (*n* = 10, 83.3%), 3 (25%) reported wounds with 6 mm in diameter and 2 (17%) showed wounds with 10 mm diameter (Table S4). Only 1 study (8%) in mice reported wounds with 4 mm, 7 mm, 12 mm, 20 mm, 40 mm diameters. Studies using rats (*n* = 2, 16.6%) presented wound sizes of 15–20 mm in diameter. Each study evaluated a different healing time, ranging from 2–14 days (Table S4).

### Characteristics of the treatments with purinergic agonists and antagonists

The concentrations of purinergic agonists administered varied widely across studies, ranging from 0.067 μg/mL to 53,700 μg/mL, reflecting different therapeutic strategies and compounds used as follows: CGS-21680 (4-[{*N*-ethyl-5*’*-carbamoyladenos-2-yl}aminoethyl] phenylpropionic acid), UTP (Uridine-5'-triphosphate), NECA (5'-N-ethylcarboxamidoadenosine), ATP (adenosine triphosphate), PDRN (polydeoxyribonucleotide), ADP (Adenosine-5'-diphosphate), TMPS (Thymidine 5'-O-monophosphorothioate) (Table S5). The doses of purinergic antagonists administered ranged from 15.4 μg/mL to 18,000 μg/mL, according to the compound used as follows: DMPX (3,7-dimethyl-1-propargylxanthine), CSC (8-(3-chlorostyryl) caffeine), SUR (Suramin), ENP (enprofylline), Caffeine, CLOP (clopidogrel), MRS 2179 (2′-deoxy-N6-methyladenosine-3′,5′-bisphosphate), and MRS 2395 (2-Chloro-3′,5′-O-(benzoyl-β,γ-methylene)adenosine 5′-triphosphate) (Table S5).

The topical route was the most commonly used (75%, *n* = 9), followed by the intraperitoneal and oral routes (8%, *n* = 1 each). Treatment duration ranged from 7 to 14 days. Purinergic agonists were applied daily in 10 studies (83%), while purinergic antagonists were administered daily in 4 studies (33%) (Table S5).

### Impact of purinergic agonists, antagonists and gene knockout for purinergic receptors on scar tissue microstructure

The histological analysis of healing tissue revealed that the activation of the purinergic pathway plays a crucial role in regulating the size and time of wound closure. In most studies, the skin repair process was accelerated by purinergic agonists, with 100% (*n* = 12) showing a reduction in healing time (Table 1, Fig. 3). Treatment with the purinergic agonists CGS-21680, UTP, NECA, PDRN, and ATP was associated with increased cell proliferation in 83% of studies (*n* = 10), as well as enhanced cell migration, suggesting that activation of purinergic receptors may contribute to these processes and potentially accelerate tissue repair. Accordingly, wounds treated with these agonists exhibited faster closure and smaller residual areas compared with untreated controls. The purinergic agonists also stimulated angiogenesis and collagenogenesis, with increased blood vessel density reported in nine studies (75%). Specifically, CGS-21680, ATP, PDRN, TMPS, and ADP upregulated tissue levels of the pro-angiogenic factor VEGF (Table 1, Fig. 3). These compounds also stimulated fibroblast activity, increasing collagen synthesis and extracellular matrix organization. Wounds treated with purinergic agonists CGS-21680, ATP, PDRN, TMPS, and ADP exhibited higher deposition of type I collagen fibers in most studies (*n* = 8, 67%), increasing scar tissue resistance and microstructural stability (Table 1, Fig. 3).

**Table 1:**
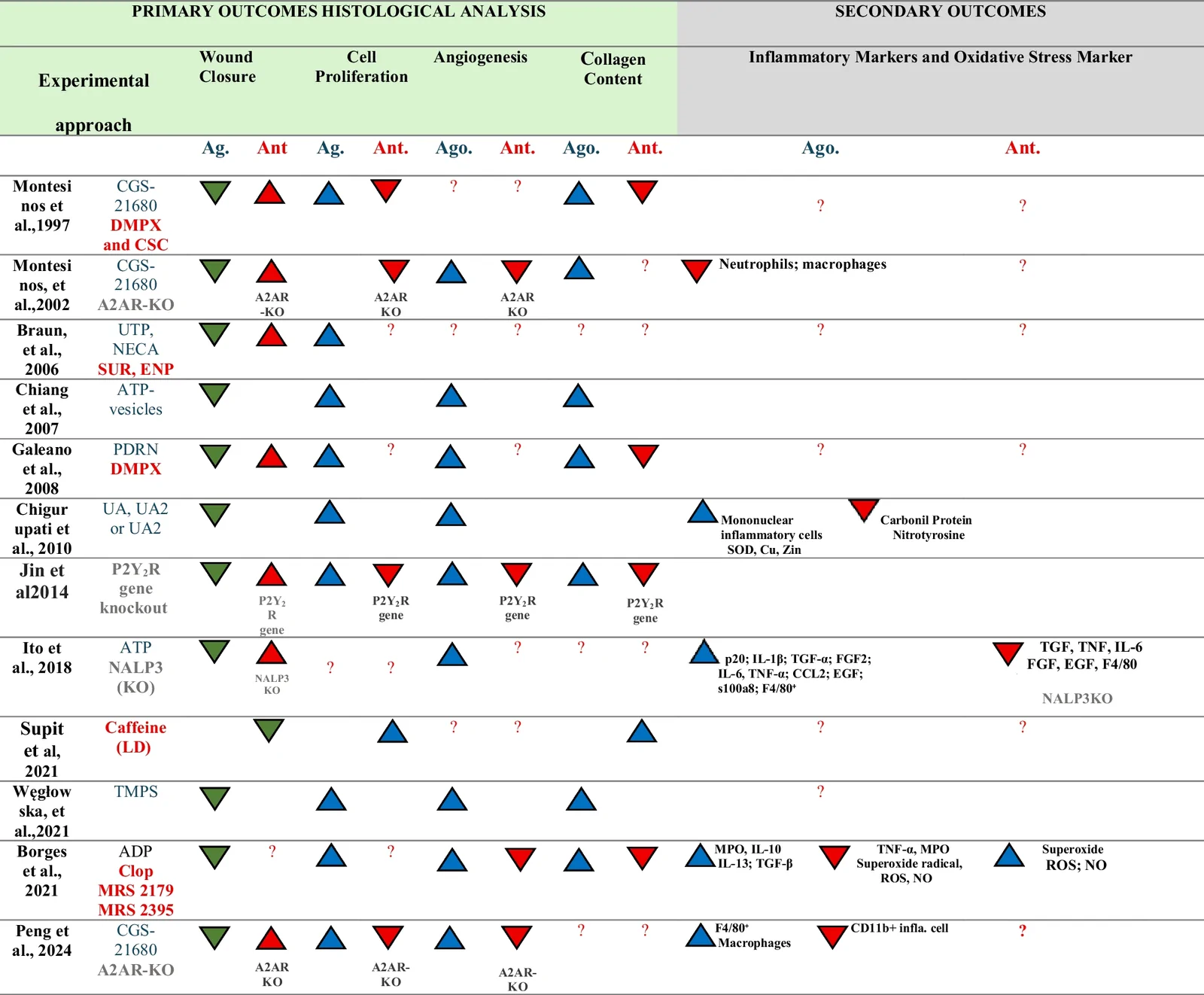
Impact of treatment with purinergic agonists (Ago.) and antagonists (Antag.) on microstructural, immunological, and oxidative parameters of scar tissue in preclinical models of skin wound healing

Green: Beneficial reduction. Blue: Increase. Red: Reduction. *Details on Healing Times, refer to supplementary material * Each study evaluated a different wound size and healing time. Caffeine induced positive and negative effects in a dose-dependent way. * Five studies evaluated the effects of antagonists. Experimental approach refers to either pharmacological modulation (agonists or antagonists) or genetic manipulation (knockout models) of purinergic signaling. Blue: Agonists (Ago.) Red: Antagonists (Antag.) Gray: KO

**Fig. 4:**
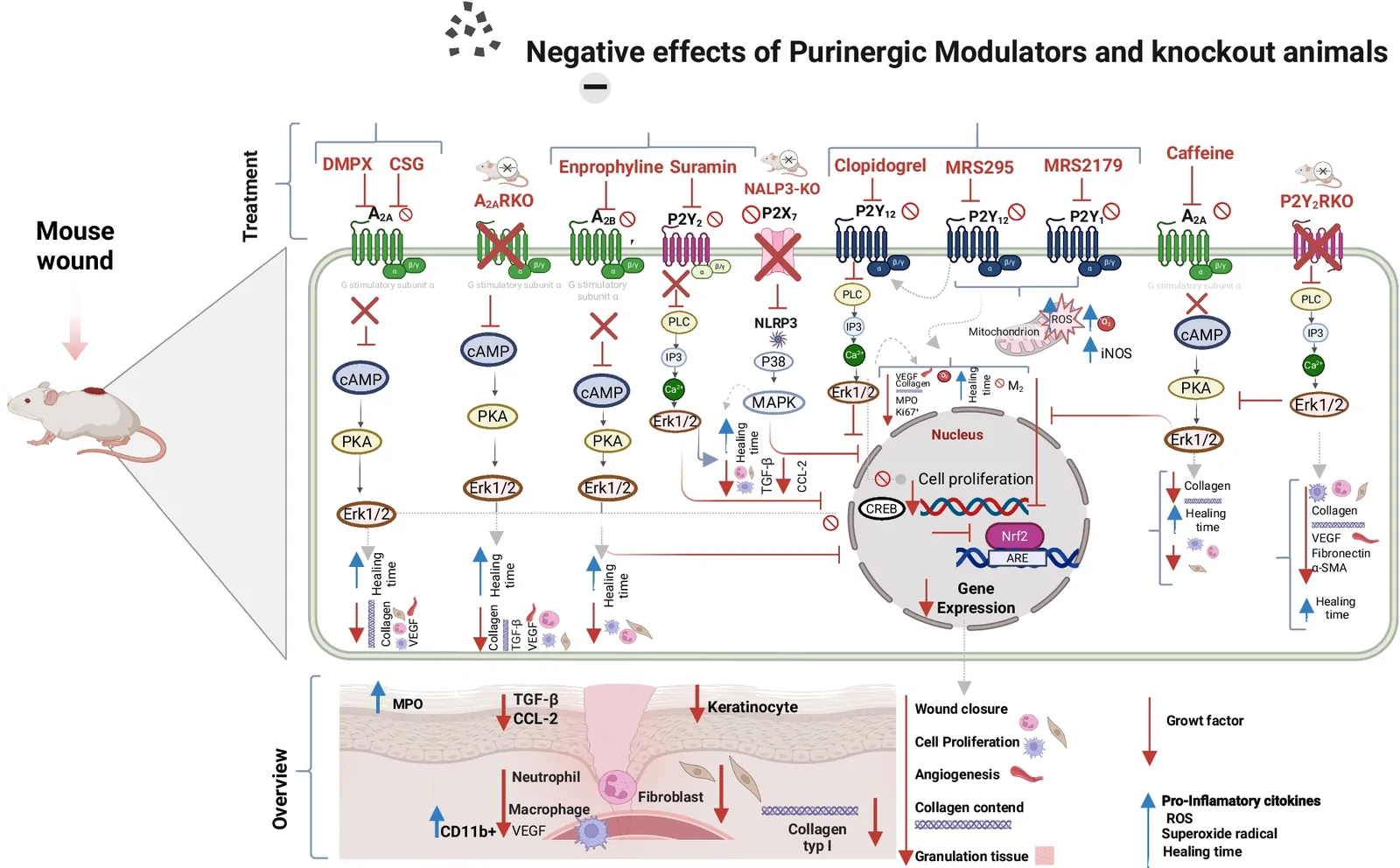
Negative effects of purinergic antagonists and receptor knockout on cutaneous wound healing. Purinergic antagonists (DMPX, CSC, SUR, ENP, caffeine, clopidogrel, MRS 2179, and MRS 2395) or purinergic receptor knockout impair wound healing by blocking P2X and P2Y receptor signalling. This inhibition disrupts MAPK and cAMP pathways, reduces NRF2 activation, and decreases VEGF expression. These changes compromise fibroblast migration, angiogenesis, collagen deposition, and extracellular matrix remodelling, delaying wound closure and impairing scar quality. In parallel, anti-inflammatory cytokines (IL-10, IL-13, TGF-β) are reduced, while pro-inflammatory cytokines (TNF-α and IL-6), MPO activity, nitric oxide, and oxidative damage are increased, resulting in sustained inflammation and defective tissue repair

In contrast, purinergic receptor antagonists were associated with detrimental effects on cutaneous wound repair. Treatment with DMPX, CSC, SUR, ENP, Clopidogrel, MRS 2179, and MRS 2395 prolonged healing time in five studies (42%). Reduced cell proliferation was also reported in five studies (42%) following antagonist administration. Attenuated angiogenesis was observed in two studies (17%) treated with DMPX and clopidogrel, while impaired collagen synthesis was reported in four studies (33%) treated with DMPX, CSC, and clopidogrel (Table 1, Fig. 4).

Although caffeine is a recognized purinergic antagonist, this systematic review demonstrated that caffeine exerted positive and negative effects on the same microstructural parameter, following a dose-dependent effect. Low-dose caffeine (19 mg/kg) improved wound healing, reduced wound contraction, and promoted favorable collagen synthesis, while higher caffeine doses (MD-HD, 37–56 mg/kg) impaired the healing process, increased wound contraction, and were associated with excessive fibroblast proliferation.

Similar to the findings observed in animals treated with purinergic antagonists, purinergic receptor knockout models exhibited impaired wound healing. Prolonged healing time was reported in A_2_A receptor knockout (A_2_AR-KO), P2Y_2_ receptor gene knockout, and NALP3-KO animals across four studies (33%). Reduced cell proliferation and angiogenesis were reported in A_2_AR-KO and P2Y_2_ receptor gene knockout models in three studies (25%). Collagen synthesis was also reduced in one study (8%) in P2Y_2_ receptor gene knockout animals (Table 1, Fig. 4).

### Impact of purinergic modulation on inflammation and oxidative stress in skin wound healing

Our results showed that the most frequently reported inflammatory parameters in the studies analyzed were inflammatory cells, cytokines, and growth factors involved in wound repair. Inflammation intensity was assessed using markers such as neutrophils, centrally recruited during early healing, and pro-inflammatory cytokines including tumor necrosis factor-α (TNF-α), interleukin-1β (IL-1β), and interleukin-13 (IL-13). Myeloperoxidase (MPO) was used as a marker of leukocyte activation, whereas interleukin-10 (IL-10) acted as an anti-inflammatory cytokine. Transforming growth factor-β (TGF-β) played a central role in regulating inflammation and promoting tissue repair. Our results showed that the purinergic agonists adenosine and ATP were associated with increased TGF-β levels in two studies (17%, *n* = 2), whereas increases in FGF2 and EGF levels were observed only in one study using ATP (8%, *n* = 1) (Table 1, Fig. 3).

Our findings indicated that purinergic the agonist CGS-21680 reduced neutrophil and macrophage recruitment in one study (8%, *n* = 1), although increased macrophage counts were also reported (8%, *n* = 1). However, one study treated with uric acid increased inflammatory cell distribution (8%, *n* = 1). In addition, treatment with the purinergic agonist adenosine was associated with TNF-α down-regulation in one study (8%, *n* = 1), whereas ATP treatment was associated with increased TNF-α and IL-1β levels in one study each (8% *n* = 1) (Table 1, Fig. 4).

Increase MPO levels were observed in one study (8%, *n* = 1) following treatment with the purinergic agonist adenosine. Our findings indicated that treatment with the purinergic agonist ATP was associated with increased interleukin-6 (IL-6) levels in one study (8%, *n* = 1), whereas adenosine treatment was associated with increased interleukin-10 (IL-10) and interleukin-13 (IL-13) levels in one study each (8%, *n* = 1) (Table 1, Fig. 3). These findings reinforce the proposition that purinergic agonists not only regulate the initial inflammation but also promote a favorable microenvironment for tissue repair, reducing the risk of chronic inflammation and aiding in the efficient remodeling of injured tissue.

Oxidative stress markers were also evaluated, especially Cu/Zn superoxide dismutase (SOD) enzyme and inducible nitric oxide synthase (iNOS). Increased SOD activity was reported in one study (8%, *n* = 1) following uric acid treatment (Table 1, Fig. 3). On the other hand, oxidative stress markers including carbonylated proteins, inducible nitric oxide synthase (iNOS), and nitrotyrosine, were reduced in one study (8%, *n* = 1) following uric acid treatment (Table 1, Fig. 3). The results showed that the purinergic agonist adenosine was associated with reduced levels of superoxide radicals, reactive oxygen species (ROS), and nitric oxide (NO) in one study (8%, *n* = 1).

Regarding treatment with the purinergic antagonist clopidogrel, reduction in myeloperoxidase (MPO) activity was reported in one study (8%) (Table 1, Fig. 4). In addition, in one study (8%), clopidogrel treatment prevented macrophage polarization toward the alternatively activated phenotype M2, characterized by increased arginase expression. Our results also indicated that clopidogrel treatment was associated with increased levels of superoxide radicals, nitric oxide synthase (NOS), and ROS in one study (8%, *n* = 1) (Table 1, Fig. 4).

In NALP3 knockout (NALP3-KO) animals, reduced levels of transforming growth factor-β (TGF-β) were reported in one study (8%), while reduced levels of fibroblast growth factor 2 (FGF2) and epidermal growth factor (EGF) were observed in one study (8%). In addition, reduced levels of CCL-2 and CD11b⁺ inflammatory cells were reported in one study with A2A receptor knockout (A2AR-KO) animals (8%, *n* = 1), as well as reduced TNF-α and IL-6 expression in one study (8%) in NALP3-KO animals (Table 1, Fig. 4).

### Risk of bias assessment

**Fig. 5:**
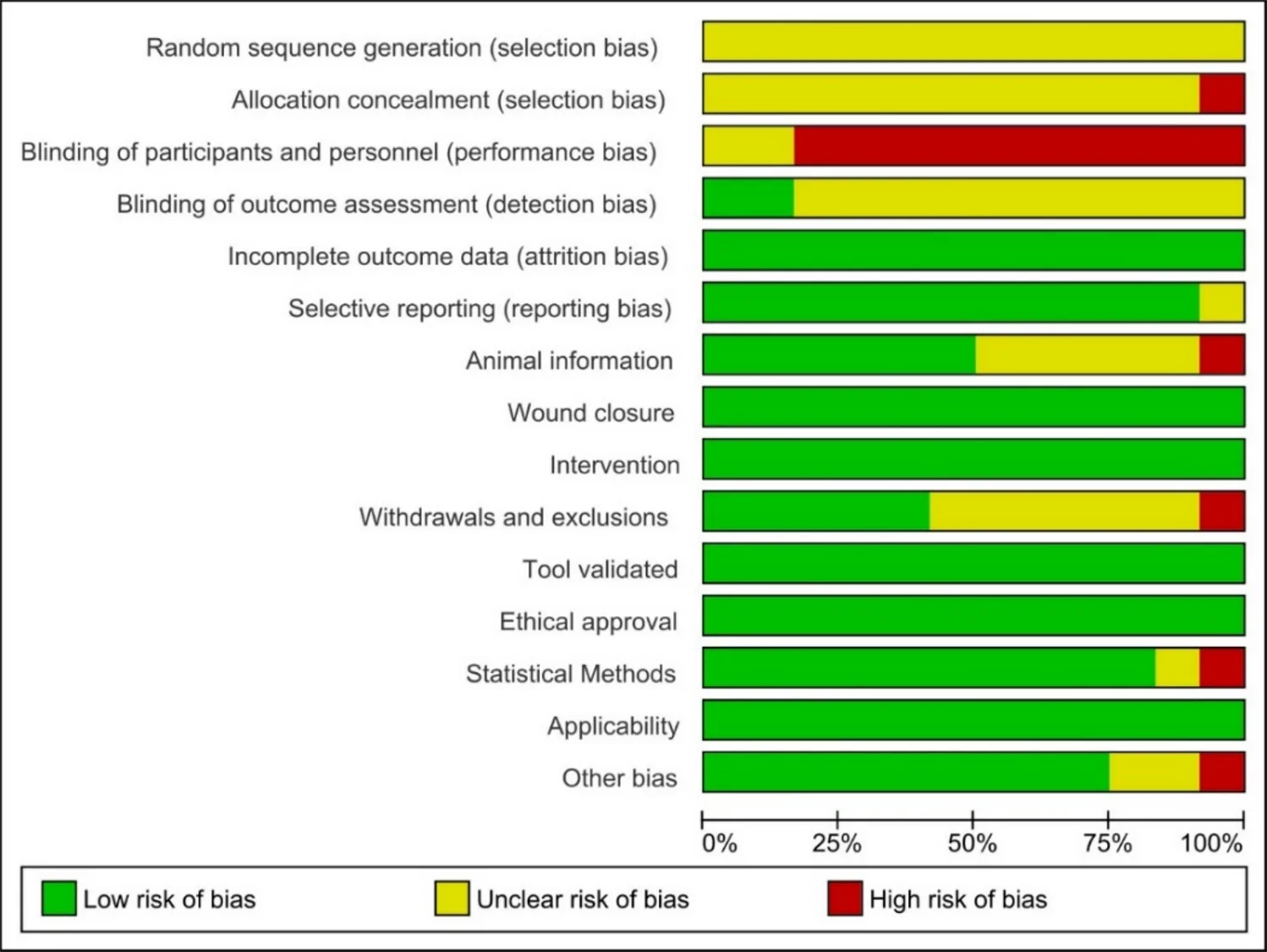
Risk of bias and methodological quality indicators for all studies included in the systematic review using the risk of bias assessment from the Systematic Review Center for Laboratory Animal Experimentation (SYRCLE). Green: indicating a low risk of bias; Red: indicating a high risk of bias; Yellow: indicating that the item was not reported, resulting in an unknown risk of bias

**Fig. 6:**
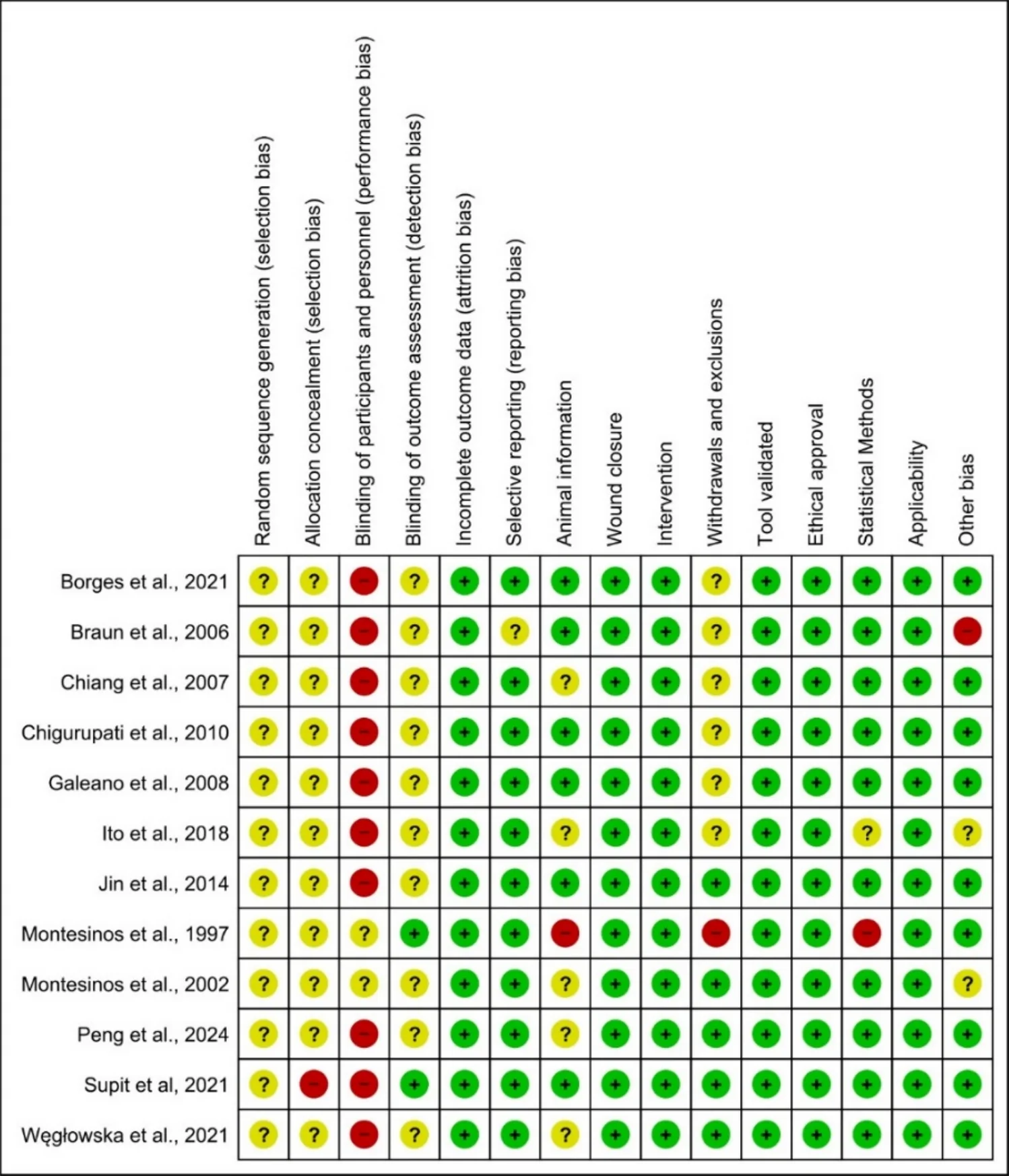
Summary of risk of bias. Analysis of the authors' judgments on the risk of bias items for each included study. Green: low risk of bias; Yellow: unclear risk of bias; and Red: high risk of bias

Detailed results for bias analysis are shown in Figs. 5 and 6. No study met all the analyzed methodological criteria. Regarding selection bias, most studies did not provide clear information on random sequence generation (100% n = 12) and allocation concealment (92%, n = 11). In terms of personnel blinding, 10 studies (83%) had a high risk of bias, while 2 (17%) had an unclear risk. For blinding of outcome assessment, 10 studies (83%) had an unclear risk, while 17% (*n* = 2) had a low risk of bias. However, a low risk of bias was found when evaluating whether studies had incomplete outcome data, 100% (n = 12) had a low risk. Regarding information on animals, 7 studies (58%) had a low risk of bias, 5 (42%) did not report this information clearly, and 1 (8%) had a high risk of bias. Additionally, wound closure had a low risk of bias in 12 studies (100%). Regarding intervention, 12 studies (100%) had a low risk of bias. Data on withdrawals and exclusions were not clearly reported in 10 studies (83%), while 1 (8%) had a low risk of bias and 1 (8%) had a high risk of bias. All studies presented a low risk of bias related to ethical approval, tool validation, and applicability. In 83% (*n* = 10) of the studies, statistical methods showed a low risk of bias, while 8% (*n* = 1) had a high risk, and 8% (*n* = 1) did not provide clear information. For applicability, 100% of the studies had a low risk of bias. Regarding other biases, 75% (*n* = 9) of the studies had a low risk, 17% (*n* = 2) had an unclear risk, and 8% (*n* = 1) had a high risk of bias.

## Discussion

### General characteristics

The effects of purinergic receptors in skin wound healing have been investigated since 1997. Our findings reveal the predominance of studies in countries such as the United States of America, followed by China, Germany, South Korea, India, Japan, Poland, and Brazil. This has likely occurred because these countries have provided strong institutional and governmental support to the field of biomedical research over the years. Specifically, the concentration of studies in the USA can be attributed to substantial investment by agencies such as the National Institutes of Health (NIH), which fund projects focused on investigating the cellular and molecular mechanisms of tissue repair, including purinergic signalling [30]. Advanced research infrastructure and internationally recognized academic centers have further facilitated these investigations. Between 1997 and 2024, there was a significant increase in studies on purinergic signalling in wound healing due to advancements in molecular biology, the discovery of purinergic receptors, and the recognition of extracellular nucleotides as key mediators in tissue repair processes [23, 31].

In addition to the United States, countries such as Germany, South Korea, India, Japan, Poland, China and Brazil have also contributed relevant publications in the field. This landscape reflects the global interest in regenerative therapies and the strengthening of biomedical research centers in these countries, driven by policies promoting scientific innovation, international collaborations, and technological advancements applied to wound healing.

### Animal models of purinergic modulation

Animal models are essential for investigating wound healing mechanisms and simulating clinical conditions [32]. Mice and rats were predominantly used due to their physiological and genetic similarities to humans, availability, and suitability for histological, macroscopic, biochemical, and biomechanical analyses across different healing phases, particularly inflammation and proliferation [32, 33]. The preference for these models is due to their greater availability, low cost, and ease of use, which contributes to increasing the reliability of the results obtained, making them a common choice in research across various fields [32, 33]. Another relevant aspect is the greater uniformity of laboratory rodents regarding experimental, environmental, and genetic variables, also considering that fewer animals are needed to conduct research effectively [32, 33].

The studies reviewed predominantly used excisional wounds in the dorsal region to investigate the main outcomes, administration methods, and the mechanisms by which purinergic pathways influence the healing of skin lesions in rats and mice. The dorsal area is easily manipulated, allowing tissue collection for the analysis of aspects such as wound contraction. Additionally, it is a location that is difficult for animals to lick when individually caged, which is advantageous since salivary glands are reservoirs of many growth factors [34].

Although the number and size of the wounds varied, the excisional model was frequently chosen due to the wound closure time, allowing for a more detailed analysis of the different phases of tissue repair, with an emphasis on the inflammatory, proliferative, and remodeling phases [32]. In the included studies, a relatively balanced distribution between sexes was observed, although with a slight predominance of male animals. However, some studies (n = 5) did not report the sex of the animals used. The choice to include both sexes suggests an attempt to broaden the applicability of the results, although in some cases males were preferred to avoid cyclical hormonal variations present in females, which can influence physiological processes involved in wound healing, such as inflammation, cell proliferation, and tissue remodelling [35].

### Intervention characteristics

Most studies used a topical administration route for purinergic modulators, allowing the activation or inactivation of purinergic receptors directly in the target tissue. This route is considered painless and less invasive, as it involves direct application to the affected area, without the need for surgical procedures or injections, minimizing discomfort and the risk of complications [36]. Intraperitoneal and oral administration were used in only one study; the intraperitoneal route was likely chosen due to difficulties in vascular access [36], while the oral route is widely used because of its simplicity, practicality, non-invasiveness. This method allows for easy administration of the treatment and is a common choice in clinical and pre-clinical studies, especially when systemic effects need to be achieved conveniently [36].

The doses of purinergic agonists and antagonists varied across studies, reflecting differences in wound size and the pharmacokinetic properties of the compounds, as well as the complexity of purinergic signalling in wound healing and the need for context-specific adjustments [37]. Most studies reported that treatment was administered daily, once a day, possibly because frequency is related to treatment duration, and the duration of the experiments was very short, typically between 10, 12, and 14 days. Daily administration likely ensures constant levels of the substance at the injury site, promoting a continuous and efficient therapeutic effect throughout the healing period. This is consistent with the dynamic and sustained role of purinergic signalling in coordinating tissue repair processes across different phases of wound healing [38].

### Impact of purinergic modulation on the scar tissue microstructure in cutaneous wounds

In this review, methodological analyses mainly focused on histological parameters, emphasizing scar tissue microstructure and inflammatory and oxidative stress markers. The reviewed studies found that purinergic receptors either assist in the healing of skin wounds or are indifferent to this process. In this context, the role of purinergic signalling stands out, gaining relevance for its ability to dynamically regulate the tissue response, acting in key steps such as cell proliferation and skin regeneration. Studies have highlighted purinergic receptors as key elements in regulating this response, making their agonists potential therapeutic targets, as the administration of exogenous nucleotides accelerates the wound healing process through the activation of specific purinergic receptors [31]. Therefore, extracellular purine nucleotides and nucleosides have biological effects on many types of cells and tissues [8].

The purinergic system consists of a signalling network involving purine-based nucleotides and nucleosides such as ATP, ADP, AMP, and adenosine in the extracellular space, which act on specific receptors known as purinoceptors, triggering a cascade of intracellular events [8, 31]. This purinergic signalling is closely related to regenerative and inflammatory processes, which makes these receptors potential therapeutic targets. In this way, purinergic modulators can positively or negatively impact scar microstructure (Figs. 3 and 4). In our studies, purinergic agonists such as ATP, ADP, CGS-21680, NECA, UTP, TMPS, and PDRN were used. In most studies, purinergic agonists induced positive effects, such as reduced wound size, increased cell proliferation, enhanced angiogenesis, and greater collagen deposition. These findings support the proposition that purinergic signalling is a promising pathway for improving wound healing efficiency and quality, potentially representing an innovative therapeutic strategy for wound treatment.

Agonists targeting adenosine receptors, particularly CGS-21680 and adenosine, increased wound closure by stimulating epithelial and fibroblast proliferation, angiogenesis, and collagen deposition via A_2_A and A2B receptor activation [8, 13]. Adenosine diphosphate (ADP) activates the P2Y_1_, P2Y_12_, and P2Y_13_ receptors, which are expressed by monocytes, macrophages, lymphocytes, mast cells, fibroblasts, keratinocytes, endothelial cells, eosinophils, platelets, neutrophils, and dendritic cells, and has been shown to increase cell proliferation [31, 39], while UTP-mediated P2Y₂ activation promoted keratinocyte and fibroblast proliferation and reepithelialization [39]. NECA-induced A₂A receptor activation stimulated angiogenesis and collagen synthesis, fundamental mechanisms for effective skin repair [13]. Corroborating our findings, P2X₇ receptor activation by ATP promoted VEGF release and angiogenesis [26], and PDRN enhanced healing through A₂A receptor activation, improving extracellular matrix remodeling and scar strength [40].

These findings support the proposition that purinergic signalling is a promising pathway to improve wound healing, representing an innovative therapeutic strategy for wound treatment. Purinergic modulators have been studied as promising agents in wound healing due to their ability to activate receptors such as P2Y_2_ and P2X, which trigger essential processes such as keratinocyte migration, fibroblast activation, neoangiogenesis, and extracellular matrix remodeling, contributing to effective reepithelialization [8]. P2X receptors belong to the class of ion channels activated by ligands, specifically activated by extracellular ATP [8]. Currently, seven distinct subtypes of this family are known. In addition, P2Y receptors belong to the superfamily of G-protein-coupled receptors, characterized by having seven transmembrane domains, with eight subtypes identified so far [8].

Upon activation, purinergic receptors trigger multiple intracellular signalling pathways, including MAPKs such as ERK1/2, p38 MAPK, and ERK/CREB signalling, which are essential for cell migration, proliferation, and angiogenesis. Evidence includes UTP-induced ERK activation via P2Y receptors [39], A₂A receptor-mediated ERK and CREB signalling [41], and adenosine-induced ERK1/2 phosphorylation, supporting the role of MAPK pathways in purinergic-mediated wound repair [39, 41].

In contrast, the studies reviewed indicated that purinergic antagonists such as DMPX, CSC, clopidogrel, MRS 2179, MRS 2395, suramin, and penprofylline exerted negative effects on skin wound repair by inhibiting the activation of several intracellular pathways, including PKA, ERK, CREB, and calcium channels. Accordingly, these compounds block purinergic receptor activation, directly impairing cell proliferation, angiogenesis, and extracellular matrix synthesis [31, 42]. As a result, we observed increased wound healing time, reduced cell proliferation, angiogenesis, and lower collagen deposition. These inhibitory effects were further corroborated by studies demonstrating impaired angiogenesis and reduced cell proliferation in response to P2Y₁₂ receptor blockade by clopidogrel, suramin treatment, and adenosine receptor antagonismo [41, 43, 44].

The importance of purinergic receptors in cutaneous healing is evidenced by studies with knockout mice (KO), where the absence of these receptors compromises essential intracellular pathways. In the case of the A₂A receptor, its deletion prevents the activation of signalling pathways such as PKA, ERK, and CREB, reducing cell proliferation, angiogenesis, collagen deposition, and delaying healing [45], while P2Y receptor deficiency compromised tissue revascularization, as observed in P2Y₂⁻/⁻ mice [46]. Additionally, other treatments such as shockwaves (which induce ATP release) showed positive results, also improved healing outcomes, although their effects cannot be attributed exclusively to purinergic signalling [19].

Based on the evidence discussed, it is clear that purinergic modulation, whether through agonists, antagonists, or knockout models, significantly influences the dynamics of cutaneous healing. While the activation of purinergic receptors promotes beneficial effects such as angiogenesis, cell proliferation, and collagen deposition, the inhibition or absence of these receptors can compromise these processes. These findings reinforce the importance of the purinergic pathway as a therapeutic target in skin wound repair.

### Impact of purinergic modulation on inflammation and oxidative stress in cutaneous wound healing

Purinergic signalling plays a central role in regulating inflammation and oxidative stress, directly influencing the wound healing process. P1 and P2 receptors activation by ATP and ADP can modulate the immune response by altering inflammatory cytokines production and the balance between reactive oxygen species and antioxidant enzymes [47, 48]. Our studies showed that treatment with the purinergic agonists ATP and ADP increased TGF-β, IL-10, and IL-13 levels. In this context, purinergic agonist administration determined a predominant anti-inflammatory effect, with increased TGF-β, IL-10, and IL-13 levels, cytokines known to promote inflammatory resolution and tissue remodeling [48]. Mechanistically, A3AR activation enhanced IL-10 production in CD4⁺ T cells [48], while P2X_7_ activation induced TGF-β1 expression via MAPK or PKC pathways [49], highlighting the immunoregulatory role of purinergic receptors.

In parallel, increased MPO levels were observed in animals treated with the purinergic agonist ADP. This finding indicates that, although these agonists have a resolving effect, they also intensify the functional activity of neutrophils during the early stages of the inflammatory response. Despite MPO upregulation, quantitative analysis revealed a reduction in the number of neutrophils in tissue treated with purinergic agonists, indicating a possible modulation in cell recruitment. These results corroborate previous studies demonstrating that purinergic signalling can regulate neutrophil activation and recruitment, promoting an environment conducive to inflammation resolution and tissue repair [50, 51].

Furthermore, reduced TNF-α levels were observed after treatment with the purinergic agonist ADP. Consistently, TNF-α levels were reduced [52], and CGS-21680-mediated activation of A_2_A and A2B receptors suppressing TNF-α production in immune cells [53], supporting the anti-inflammatory profile of purinergic signalling [47, 50]. Thus, TNF-α down-regulation reinforces the role of purinergic agonists in therapeutic strategies to modulate inflammation and accelerate the healing process.

Purinergic agonists also improved redox homeostasis by increasing Cu/Zn superoxide dismutase (SOD) activity, particularly following treatment with 6,8-dithiouric acid [54]. This effect was associated with activation of the Nrf2 pathway, a central regulator of antioxidant responses in wound healing [55]. Monosodium urate crystals and ADP-induced P2Y_13_ activation promoted Nrf2 nuclear translocation and expression of antioxidant enzymes such as heme oxygenase-1 (HO-1) [55]. P1 and P2Y receptors activation consistently reduced ROS production and oxidative tissue damage [56, 57], as reflected by lower levels of carbonylated proteins, iNOS, nitrotyrosine, and ROS, preserving tissue integrity. Models treated with uric acid and ADP also showed a decrease in classical oxidative stress markers, including carbonyl proteins, iNOS, nitrotyrosine, and ROS levels (54, 3), reinforcing the role of purinergic signalling in protecting against cellular dysfunction. Together, these results support the hypothesis that purinergic signalling plays a multifaceted role in the inflammatory microenvironment. In addition to modulating the activation and recruitment of immune cells, purinergic modulators contribute to oxidative stress control. This dual action creates a biochemical environment favorable to tissue regeneration, highlighting the therapeutic potential of purinergic agonists in the treatment of chronic injuries or persistent inflammatory processes.

In contrast, treatment with specific purinergic receptor antagonists resulted in impaired wound healing, with negative effects on the inflammatory and oxidative environments, although some inflammatory markers were reduced while oxidative markers remained upregulated (Fig. 4). Our findings showed a reduction in MPO levels after treatment with the purinergic antagonist clopidogrel. Purinergic receptor blockade reduced MPO activity, indicating compromised neutrophil activation [51], and inhibited macrophage polarization toward the M2 phenotype, disrupting inflammation resolution. Activation of P2Y_2_ and A_2_A receptors is essential for anti-inflammatory and reparative responses [58], while adenosine promotes M2 macrophage differentiation [58], effects abolished by receptor inhibition. Therefore, the fact that blocking these receptors inhibited M2 macrophages polarization reinforces the essential role of purinergic signalling in the transition from inflammation to the resolution phase. Moreover, clopidogrel increased superoxide radicals, NOS, and ROS levels, consistent with studies demonstrating that inhibition of this purinergic signalling pathway may exacerbate oxidative tissue damage [38, 59]. These results reinforce that purinergic signalling inhibition impairs inflammation modulation and redox balance, compromising the healing process.

Our findings demonstrated reduced TGF-β, EGF, and FGF levels in knockout animals, corroborating previous studies reporting that reduced TGF-β levels impaired fibroblast activation and extracellular matrix deposition [60], while the absence of P2Y2 receptors compromised tissue regeneration [60]. These findings reinforce the idea that the interaction between components of purinergic signalling with inflammatory and redox effectors appears to be crucial for the proper resolution of the wound healing process.

### Limitations

Using the SYRCLE tool, specific limitations in the research reports were identified, where we found that most studies did not clearly indicate important methodological details, such as random sequence generation, random outcome assessment, and participant blinding. In addition, the heterogeneity of the studies reviewed was an important limitation identified, which makes it difficult a more objective comparative analysis. The lack of information about animals sex and weight was also overlooked in some studies, which may introduce reporting bias, as it compromises the quality of the scientific report. Thus, it is relevant to highlight that our findings are significant for understanding the mechanism of purinergic signalling in wound healing, describing important sources of bias. Additionally, this review was restricted to mammalian preclinical models, particularly mice and rats, due to their greater physiological and immunological similarity to human skin. Therefore, although the present review focused on rodent models, different regenerative models (e.g., zebrafish) may provide complementary insights into purinergic signalling and tissue regeneration and could be explored in further investigations. Therefore, we hope that this systematic review, while acknowledging its intrinsic qualitative nature in describing key bias points, will contribute to future studies by reporting elements of bias that undermine the quality of evidence and that should be overcome through investigations with greater methodological rigor.

## Conclusion

Based on the studies included in this systematic review, modulation of the purinergic signalling pathway through agonists and antagonists of the purinergic receptors A_2_A, P2X_7_, P2Y_2_, P2Y_6_, and P2Y_12_, as well as the functional absence of these receptors in murine knockout models, was associated produced distinct effects on cutaneous wound healing outcomes. Activation of these receptors by the agonists CGS-21680, NECA, UTP, PDRN, ATP, ADP, and TMPS was associated with improved wound repair parameters, increased fibroblast and keratinocyte proliferation, enhanced angiogenesis, and increased collagen biosynthesis. These effects were accompanied by modulation of the Nrf2, MAPK, and ERK/CREB molecular pathways, accompanied by reduced inflammatory infiltrate and TNF-α levels, decreased markers of oxidative and nitrosative stress, and increased production of regulatory and anti-inflammatory cytokines (IL-10, IL-13, and TGF-β), as well as growth factors, such as FGF and EGF. Conversely, inhibition of A_2_A, P2Y_1_, P2Y_2_, and P2Y_12_ receptors by the antagonists DMPX, CSC, enprofylline, suramin, clopidogrel, MRS 2179, MRS 2395, and high-dose caffeine was generally associated with delayed wound closure, reduced cell proliferation, angiogenesis, and collagen deposition, as well as suppression of Nrf2, MAPK, and ERK/CREB signalling. Complementarily, murine knockout models for the A_2_A and P2Y2 receptors exhibited responses consistent with those observed in antagonist-treated studies, suggesting an important role of these purinergic receptors in regulating the cellular and molecular events involved in cutaneous wound healing.

Taken together, the results of this review indicate that selective activation of the purinergic receptors A_2_A, P2Y_2_, P2X_7_, P2Y_6_, and P2Y_12_ may contribute with an improved cutaneous wound healing response, whereas inhibition or functional absence of the A_2_A, P2Y_1_, P2Y_2_, and P2Y_12_ receptors may impair this process. The evidence synthesized in this systematic review supports the biotechnological potential of purinergic modulators as possible therapeutic targets for the development of novel strategies for skin wound repair, as selective activation of specific purinergic receptors was consistently associated with improved cutaneous wound healing outcomes. However, the available evidence remains limited to preclinical studies, predominantly conducted in murine models, with considerable methodological heterogeneity among studies. Therefore, further well-designed preclinical and translational studies are required to confirm their therapeutic applicability and mechanistic effects in tissue regeneration.

## Supplementary Information

Below is the link to the electronic supplementary material.

Supplementary file1 (DOCX 227 KB)

## Data Availability

All data used in the article are available in the supplementary file and are described in the reviewed articles.

## Abbreviations

*ADP*: Adenosine-5′-diphosphate
*AMP*: Adenosine Monophosphate
*ATP*: Adenosine triphosphate
*Ca*^2^⁺: Calcium ion
*CD4⁺ T cells*: Cluster of Differentiation 4 Positive T Lymphocytes
*CGS-21680*: 4-[{N-ethyl-5-(9-carbamoyladenos-2-yl)aminoethyl}] phenylpropionic acid
*Cu/Zn SOD*: Copper/Zinc Superoxide Dismutase
*CLOP*: Clopidogrel
*CSC*: 8-(3-Chlorostyryl) caffeine
*DAMPs*: Damage-Associated Molecular Patterns
*DMPX*: 3,7-Dimethyl-1-propargylxanthine
*EGF*: Epidermal Growth Factor
*ENP*: Enprofylline
*ERK*: Extracellular Signal-Regulated Kinase
*FGF*: Fibroblast Growth Factor
*HO-1*: Heme Oxygenase-1
*IL-10*: Interleukin-10
*IL-13*: Interleukin-13
*K⁺*: Potassium ion
*KO*: Knockout
*MAPK*: Mitogen-Activated Protein Kinase
*MPO*: Myeloperoxidase
*MRS 2179*: 2′-Deoxy-N6-methyladenosine-3′,5′-bisphosphate
*MRS 2395*: 2-Chloro-3′,5′-O-(benzoyl-β,γ-methylene) adenosine-5′-triphosphate
*Na⁺*: *Sodium ion*
*NECA*: 5′-N-ethylcarboxamidoadenosine
*Nrf2*: Nuclear factor erythroid 2–related factor 2
*NOS*: Nitric Oxide Synthase
*PAMPs*: *Pathogen-Associated Molecular Patterns*
*PDRN*: Polydeoxyribonucleotide
*ROS*: Reactive Oxygen Species
*SUR*: Suramin
*SYRCLE*: Systematic Review Centre for Laboratory Animal Experimentation
*TGF-β*: Transforming Growth Factor-beta
*TMPS*: Thymidine 5′-O-monophosphorothioate
*TNF-α*: Tumor Necrosis Factor Alpha
*UTP*: Uridine-5′-triphosphate
