# Supplementary material for: Interaction between purinergic signalling and purinergic modulators with skin wound repair effectors: a systematic review of preclinical in vivo models
Source: Purinergic Signal. 2026 Aug 31;22(5):67. doi: 10.1007/s11302-026-10178-y (PMC13530080; doi:10.1007/s11302-026-10178-y)
Supplement: Supplementary file 1 [file 11302_2026_10178_MOESM1_ESM.docx]

**Supplementary Material**

**Table S1:** Full search strategy in PubMed, Scopus, Web of Science, Embase and CINAHL including search terms and filters.

**Table S2:** Description of the studies included in this systematic review that evaluated purinergic signaling in wound healing in murine models.

**Table S3.** Animal model Characteristics.

**Table S4.** Qualitative summary with details on the efficacy of purinergic agonist treatment on wound healing.

**Table S5.** Intervention characteristic purinergic signalling: agonists, antagonists, and knockout models.

**Figure S1. Countries included in this Review.** Distribution of countries included in this systematic review. The places where the studies were conducted were reported in all studies reviewed. Colors indicate the relative contribution of each country, highlighting the predominance of studies from the United States, followed by Brazil, China, Germany, India, Japan, Poland, and South Korea.

**Supplementary material**

**Table S1:** Full search strategy used in PubMed, Scopus, Web of Science, Embase and Cinahl (EBSCO) including search descriptors, research records and date.

| **Database** | **Research descriptors** | **Research records (n)** | **Date and time** |
| --- | --- | --- | --- |
| **PubMed** | Purinergic signaling[TIAB] OR Purinergic receptor*[TIAB] OR Adenosine receptor*[TIAB] OR Adenosine diphosphate[TIAB] OR Adenosine triphosphate[TIAB] OR Purinergic Agonist*[TIAB] OR Purinergic Antagonists*[TIAB] OR Purine*[TIAB] OR Adenine[TIAB] OR Guanine[TIAB] OR hypoxanthine[TIAB] OR xanthine[TIAB] OR theobromine[TIAB] OR caffeine[TIAB] OR uric acid[TIAB] OR isoguanine[TIAB] OR [TIAB]) AND (Wound Healing*[TIAB]) AND (Skin[TIAB] OR Dermis[TIAB] OR Granulation tissue[TIAB] OR Epidermis[TIAB] OR Keratinocytes[TIAB] OR Integumentary system OR Dermatology[TIAB] OR Dermoscopy[TIAB] OR Wounds and Injuries[TIAB] OR Fibrosis[TIAB] OR Scar*[TIAB] OR Cicatrix[TIAB]) | 62 | 09/12/24  17:02 PM |
| **SCOPUS** | (TITLE-ABS-KEY("Purinergic signaling" OR "Purinergic receptor*" OR "Adenosine receptor*" OR "Adenosine diphosphate" OR "Adenosine triphosphate" OR "Purinergic Agonist*" OR "Purinergic Antagonists*" OR "Purine*" OR "Adenine" OR "Guanine" OR "hypoxanthine" OR "xanthine" OR "theobromine" OR "caffeine" OR "uric acid" OR "isoguanine") AND TITLE-ABS-KEY("Wound Healing*") AND TITLE-ABS-KEY("Skin" OR "Dermis" OR "Granulation tissue" OR "Epidermis" OR "Keratinocytes" OR "Integumentary system" OR "Dermatology" OR "Dermoscopy" OR "Wounds and Injuries" OR "Fibrosis" OR "Scar*" OR "Cicatrix") | 321 | 10/12/24  08:43 AM |
| **Web of Science** | TS=("Purinergic signaling" OR "Purinergic receptor*" OR "Adenosine receptor*" OR "Adenosine diphosphate" OR "Adenosine triphosphate" OR "Purinergic Agonist*" OR "Purinergic Antagonists*" OR "Purine*" OR "Adenine" OR "Guanine" OR "hypoxanthine" OR "xanthine" OR "theobromine" OR "caffeine" OR "uric acid" OR "isoguanine") AND TS=("Wound Healing*") AND TS= ("Skin" OR "Dermis" OR "Granulation tissue" OR "Epidermis" OR "Keratinocytes" OR "Integumentary system" OR "Dermatology" OR "Dermoscopy" OR "Wounds and Injuries" OR "Fibrosis" OR "Scar*" OR "Cicatrix") | 208 | 10/12/24  09:29 AM |

**Table S1 (*continuation*): Table S1:** Full search strategy used in PubMed, Scopus, Web of Science, Embase and Cinahl (EBSCO) including search descriptors, research records and date.

| **Database** | **Research descriptors** | **Research records (n)** | **Date and time** |
| --- | --- | --- | --- |
| **Embase** | (purinergic signaling/exp OR ‘Purinergic receptor’ OR ‘Adenosine receptor’ OR ‘Adenosine diphosphate’ OR ‘Adenosine triphosphate’ OR ‘Purinergic Agonist’ OR ‘Purinergic Antagonists’ OR ‘Purine’ OR ‘Adenine’ OR ‘Guanine’ OR ‘hypoxanthine’ OR ‘xanthine’ OR ‘theobromine’ OR ‘caffeine’ OR ‘uric acid’ OR ‘isoguanine’) AND (Wound Healing/exp) AND (Skin/exp OR ‘Dermis’ OR ‘Granulation tissue’ OR ‘Epidermis’ OR ‘Keratinocytes’ OR ‘Integumentary system’ OR ‘Dermatolog’ OR ‘Dermoscopy’ OR ‘Wounds’) AND (‘Injuries’ OR ‘Fibrosis’ OR ‘Scar’ OR ‘Cicatrix’) | 116 | 31/01/25  22:30 PM |
| **Cinahl (EBSCO)** | (AB"Purinergic signaling" OR AB"Purinergic receptor*" OR AB"Adenosine receptor*" OR AB"Adenosine diphosphate" OR AB"Adenosine triphosphate" OR AB"Purinergic Agonist*" OR AB"Purinergic Antagonists*" OR AB"Purine*" OR AB"Adenine" OR AB"Guanine" OR AB"hypoxanthine" OR AB"xanthine" OR AB"theobromine" OR AB"caffeine" OR AB"uric acid" OR AB"isoguanine") AND (AB"Wound Healing") AND (AB"Skin") | 149 | 03/04/25  10:00 AM |

**Table S2:** Studies included in the systematic review evaluating the impact of purinergic signaling modulation in murine model of skin wound healing.

|  | **Complete reference** | **Country** |
| --- | --- | --- |
| 1 | Montesinos MC, Gadangi P, Longaker M, Sung J, Levine J, Nilsen D, Reibman J, Li M, Jiang CK, Hirschhorn R, Recht PA, Ostad E, Levin RI, Cronstein BN. Wound healing is accelerated by agonists of adenosine A2 (G alpha s-linked) receptors. J Exp Med. 1997 Nov 3;186(9):1615-20. PMID: 9348321; PMCID: PMC2199104. | USA |
| 2 | Montesinos MC, Desai A, Chen JF, Yee H, Schwarzschild MA, Fink JS, Cronstein BN. Adenosine promotes wound healing and mediates angiogenesis in response to tissue injury via occupancy of A(2A) receptors. Am J Pathol. 2002 Jun;160(6):2009-18. PMID: 12057906; PMCID: PMC1850820. | USA |
| 3 | Braun M, Lelieur K, Kietzmann M. Purinergic substances promote murine keratinocyte proliferation and enhance impaired wound healing in mice. Wound Repair Regen. 2006 Mar-Apr;14(2):152-61. PMID: 16630104. | Germany |
| 4 | Chiang B, Essick E, Ehringer W, Murphree S, Hauck MA, Li M, Chien S. Enhancing skin wound healing by direct delivery of intracellular adenosine triphosphate. Am J Surg. 2007 Feb;193(2):213-8. Erratum in: Am J Surg. 2008 Jan;195(1):139. PMID: 17236849; PMCID: PMC1850226. | USA |
| 5 | Galeano M, Bitto A, Altavilla D, Minutoli L, Polito F, Calò M, Lo Cascio P, Stagno d'Alcontres F, Squadrito F. Polydeoxyribonucleotide stimulates angiogenesis and wound healing in the genetically diabetic mouse. Wound Repair Regen. 2008 Mar-Apr;16(2):208-17. PMID: 18318806. | USA |
| 6 | Chigurupati S, Mughal MR, Chan SL, Arumugam TV, Baharani A, Tang SC, Yu QS, Holloway HW, Wheeler R, Poosala S, Greig NH, Mattson MP. A synthetic uric acid analog accelerates cutaneous wound healing in mice. PLoS One. 2010 Apr 6;5(4):e10044. PMID: 20386608; PMCID: PMC2850366. | USA |
| 7 | Jin H, Seo J, Eun SY, Joo YN, Park SW, Lee JH, Chang KC, Kim HJ. P2Y2 R activation by nucleotides promotes skin wound-healing process. Exp Dermatol. 2014 Jul;23(7):480-5. PMID: 24816122. | Korea |
| 8 | Ito H, Kanbe A, Sakai H, Seishima M. Activation of NLRP3 signalling accelerates skin wound healing. Exp Dermatol. 2018 Jan;27(1):80-86. Epub 2017 Nov 2. PMID: 28887870. | India |
| 9 | Supit T, Susilaningsih N, Prasetyo A; Najatullah. Effects of Caffeine Consumption on Autologous Full-Thickness Skin Graft Healing in an Animal Model. Indian J Plast Surg. 2021 Sep 16;54(3):314-320. PMID: 34667517; PMCID: PMC8515418. | Japan |
| 10 | Węgłowska E, Koziołkiewicz M, Kamińska D, Grobelski B, Pawełczak D, Kołodziejczyk M, Bielecki S, Gendaszewska-Darmach E. Extracellular Nucleotides Affect the Proangiogenic Behavior of Fibroblasts, Keratinocytes, and Endothelial Cells. Int J Mol Sci. 2021 Dec 27;23(1):238. PMID: 35008664; PMCID: PMC8745609. | Poland |
| 11 | Borges PA, Waclawiak I, Georgii JL, Fraga-Junior VDS, Barros JF, Lemos FS, Russo-Abrahão T, Saraiva EM, Takiya CM, Coutinho-Silva R, Penido C, Mermelstein C, Meyer-Fernandes JR, Canto FB, Neves JS, Melo PA, Canetti C, Benjamim CF. Adenosine Diphosphate Improves Wound Healing in Diabetic Mice Through P2Y_12_ Receptor Activation. Front Immunol. 2021 Mar 22; 12:651740. PMID: 33828561; PMCID: PMC8019717. | Brazil |
| 12 | Peng Y, Xiong R, Wang B, Chen X, Ning Y, Zhao Y, Yang N, Zhang J, Li C, Zhou Y, Li P. The Essential Role of Angiogenesis in Adenosine 2A Receptor Deficiency-mediated Impairment of Wound Healing Involving c-Ski via the ERK/CREB Pathways. Int J Biol Sci. 2024 Aug 19;20(11):4532-4550. PMID: 39247808; PMCID: PMC11380447. | China |

**Table S3.** Characteristics of animal model used to investigate the impact of purinergic signaling modulation on skin wound healing.

| **Study** | **Animal species** | **Lineage** | **Sex** | **Age**  **(week-old)** | **Weight (g)** | **Animals (n)** | **Knockout** | **Animals/ group** |
| --- | --- | --- | --- | --- | --- | --- | --- | --- |
| Montesinos et al.,1997 | Mice | BALB/c | Male and Female | 6–8 | ? | 36 | No | 10 /3 |
| Montesinos et al., 2002 | Mice | BALB/c | Female and Male | 12-15 | ? | 70 | Yes | 19/4 |
| Braun, et al., 2006 | Mice | NMRI mice | Female | ? | 25 | 36 | No | 6 /6 |
| Chiang et al., 2007 | Mice | Nude mice | ? | ? | 20-30 | 52 | No | 26/2 |
| Galeano et al., 2008 | Mice | C57BL/KsJ | Female | 14 | 22–24 | 84 | No | 21/4 |
| Chigurupati et al., 2010 | Mice | C57BL/6 | Male | 12-16 | ? | 12 | No | 6 / 2 |
| Jin et al., 2014 | Mice | C57BL/6 | ? | 6 | ? | 18 | Yes | 9/2 |
| Ito et al., 2018 | Mice | C57BL/6J | ? | 8-10 | ? | 24 | Yes | 5/2 |
| Supit et al, 2021 | Rats | Sprague–Dawley | Male | 13-15 | 200 | 40 | No | 10/4 |
| Węgłowska et al.,2021 | Rats | Wistar | ? | 8-13 | ? | 12 | No | 6 /2 |
| Borges et al., 2021 | Mice | Swiss | Male | 8-10 | 25±30 | 40 | No | 8 /5 |
| Peng et al., 2024 | Mice | C57BL/6 | ? | 13 | ? | 12 | Yes | 6/ 2 |

? = Data not reported in the article.

|  | **Table S4. Qualitative Summary with Details on the Efficacy of Purinergic Agonist Treatment on Wound Healing** | | | | | | |
| --- | --- | --- | --- | --- | --- | --- | --- |
| **Study** | **Wound Size** | **Wound Closure Time** | **Cell Proliferation** | **Angiogenesis** | **Collagen Content** | **Inflammatory**  **Markers** | **Oxidative Stress Marker** |
| Montesinos et al.,1997 | 12 mm  Excisional wound- dorsal | Treated Group :CGS-21680: 2 days: 50% wound closure  Control Group: 6 days = 50% wound closure | Fibroblasts |  | Extracellular matrix density |  |  |
| Montesinos, et al.,2002 | 10 mm  Excisional wound dorsal | Treated Group: CGS-21680 (WT)- 11 a 12 days - 50% closure  A2AKO: 12 a 13 days: 50% wound closure | Fibroblasts | WT: angiogenesis, endothelial cells, vascularization  A2AKO: Microvessel formation | Fibroblasts  Matrix density and reepithelialization | Neutrophils and Macrophages  Leukocytes |  |
| Braun, et al., 2006 | 6 mm  Excisional wound dorsal | Treated Group :NECA: 10days- wound área: 3,6 ± 1,5% Treated Group UTP:10 days: wound área: 3,9 ± 1,8%  Control Group :10 days: wound área: 18,4 ± 6,6% | Keratinocytes  P2Y |  |  |  |  |
| Chiang et al., 2007 | 6 mm  Excisional wound head | Treated group: ATP: wound área: 12,27 ± 0,48 dia  Control Group: wound área: 16,91 ± 0,87 dias | Keratinocytes | VEGF expression | Treated group: ATP: Granulation Tissue    Control Group: Tecido de granulação |  |  |
| Galeano et al., 2008 | 40mm  Excisional wound intraperitoneal | Treated group: PDRN: 12 days- Complete reepithelialization  Control Group: incomplete reepithelialization on day 12. | Cell proliferation  Fibroblasts  Endothelial cells | VEGF, CD31 between 3 to 6 days,  angiopoietin-1 | Collagen deposition and tissue organization, transglutaminase-II, and fibroblast-rich granulation tissue | - |  |
| Chigurupati et al., 2010 | 4mm  Excisional wound dorsal | Treated group :UA2: 8 days> restauração da derme e epiderme   Control Group: incomplete reepithelialization on day 13. | Cell proliferation  e diferenciação de miofibroblastos | Angiogenesis; blood vessels |  | Infiltrated mononuclear inflammatory cells | SOD Cu/Zn  Carbonylated Protein |
| Jin et al., 2014 | 7 mm  Excisional wound dorsal | WT:wound closure A2AKO | Ki67 (WT)  Ki67 (A2KO) | WT: VEGF  A2AKO: VEGF | WT: Type III collagen fibers  A2AKO: Type III collagen fibers |  |  |
| Ito et al., 2018 | 6 mm  Excisional wound dorsal | WT: wound closure: 6 days  A2AKO: wound healing delay |  | WT: VEGF  A2AKO: VEGF |  | WT: p20 e IL-1β, FGF2 EGF e TGF-α, IL-6 e s100a8   A2AKO: TGF-α IL-6 |  |
| Supit et al, 2021 | 15 mm  Excisional wound  gavage oral | LD Caffeine) HD and MD Caffeine) | Fibroblasts (LD) Fibroblasts (HD, MD) |  | Collagen deposition HD AND MD Caffeine)  (LD Caffeine) |  |  |
| Węgłowska, et al.,2021 | 20mm  Incisional wound dorsal | Wound healing evaluation date (30 days after the implantation of BNC biomaterial soaked in TMPS) | Cell proliferation Fibroblasts | VEGF, blood vessels, and capillaries | Extracellular matrix remodeling,  MMP2, MACF1 |  |  |
| Borges et al., 2021 | 10 mm  Excisional wound dorsal | Treated group ADP: 60% wound closure: 7 dias, 14 days: Complete healing  Control Group: 2% wound closure: 7days | Ki67  Fibroblasts |  | Collagen deposition. Type III collagen to Type I collagen. α-SMA expression | MPO, Neutrophils  IL-10, TGF-β, IL-13  TNF-α | Superoxide Radical  ROS, NO |
| Peng et al., 2024 | 20mm  Excisional wound buttocks) | WT- Treated group: 9 days - Expression of A2AR in the epidermis and granulation tissue in the wound knockout A2AKO: wound healing delay | Cell proliferation   c-Ski, Fibroblasts | WT: CD3 endothelial cells, Expression of c-Ski  A2AKO: Angiogenesis |  | Macrófagos F4/80⁺.  CD11b+ Inflammatory cells |  |

**Table S5.** Intervention characteristic purinergic signalling: agonists, antagonists, and knockout models.

| **References** | **Intervention name** | **Chemical name / Gene knockout** | **Dose (μg/mL)** | **Administration route** | **Duration (day)** | **Control** |
| --- | --- | --- | --- | --- | --- | --- |
| Montesinos et al.,1997 | CGS-21680  DMPX and CSC | 4- {N-ethyl-5 9-carbamoyladenos-2-yl} aminoethyl] phenylpropionic acid)  3,7-dimethyl-1-propargylxanthine  CSC (8-(3-chlorostyryl) caffeine) | CGS-21680: 250  DMPX: 2500 CSC: 250 | Topical | 1x day / 10 days | 1.5%, wt/vol  carboxymethylcellulose in PBS |
| Montesinos et al.,2002 | CGS-21680 | 2- p- [2-carboxietil] fenetil-amino-5' -N -etilcarboxamido-adenosina | 250 | Topical | 1x day / 14 days | 1.5% w/v carboxymethylcellulose in phosphate-buffered  saline (PBS) |
| Braun et al., 2006 | UTP, NECA  SUR, ENP | NECA[5'-N-ethylcarboxamidoadenosine] UTP [Uridine-5'-triphosphate]  SUR(Suramin) and ENP (enprofylline) | UTP- 484.14  NECA- 53700  SUR-1297  ENP - 18000 | Topical | 1x day / 10 days | Dexamethasone  + vehicle |
| Chiang et al., 2007 | ATP-vesicles | Adenosine triphosphate | 12.68 | Topical | 1x day / 12 days | Lipid vesicles |
| Galeano et al., 2008 | PDRN  DMPX | Polydeoxyribonucleotide  3,7-dimetil-1-propargilxantina | PDRN: 1840  DMPX: 23 | Intraperitoneal | 1x day / 12 days | Saline solution |
| Chigurupati et al., 2010 | UA, UA2 or UA2 | 6, 8 dithio-uric acid (UA2) 1, 7 dimethyl-UA | 22 | Topical | 1x day / 13 days | Vehicle |
| Jin et al., 2014 | P2Y_2_R gene knockout | Gene knockout | ? | ? | 1x day / 14 days | P2Y_2_R  knockout |
| Ito et al., 2018 | ATP | Adenosine triphosphate | 500 | Topical | Once every other day /3 days | Vehicle (White petrolatum) |
| Supit et al., 2021 | Caffeine | Caffeine | 19 (LD)  37 (MD)  56(HD) | Gavage oral | 1x day / 7 days | Drinking water |
| Węgłowska et al.,2021 | TMPS- agonista do receptor P2Y | Thymidine 5′-O-monophosphorothioate | 2333 | Topical | Single dose | PBS |
| Borges et al., 2021 | ADP  Clop  MRS 2179  MRS 2395 | Adenosine-5'-diphosphate (ADP)   Clopidogrel  MRS 2179: 2′-deoxy-N6-methyladenosine-3′,5′-bisphosphate  MRS 2395: 2-Chloro-3′,5′-O-(benzoyl-β,γ-methylene)adenosine 5′-triphosphate | ADP- 15.2  Clop - 150  MRS 2179: 15.4 MRS 2395- 17.2 | Topical | 1x day / 14 days | Saline |
| Peng et al., 2024 | CGS-21680 | 4- [{N-ethyl-5 9-carbamoyladenos-2-yl} aminoethyl] phenylpropionic acid) | 0.067 μg/mL | Topical | Single dose | DMSO |

? = Data not reported in the article.

**Figure S1.** Countries included in this Review

**Figure S1. Countries included in this Review.** Distribution of countries included in this systematic review. The places where the studies were conducted were reported in all studies reviewed. Colors indicate the relative contribution of each country, highlighting the predominance of studies from the United States, followed by Brazil, China, Germany, India, Japan, Poland, and South Korea.
